# Validation of the Clinical Index of Stable Febrile Neutropenia (CISNE) model in febrile neutropenia patients visiting the emergency department. Can it guide emergency physicians to a reasonable decision on outpatient *vs*. inpatient treatment?

**DOI:** 10.1371/journal.pone.0210019

**Published:** 2018-12-31

**Authors:** Hae Moon, Young Ju Choi, Sung Hoon Sim

**Affiliations:** 1 Department of Internal Medicine, National Cancer Center, Goyang, Gyeonggi-do, Republic of Korea; 2 Infectious Diseases Clinic, National Cancer Center, Goyang, Gyeonggi-do, Republic of Korea; 3 Center for Breast Cancer, National Cancer Center, Goyang, Gyeonggi-do, Republic of Korea; 4 Translational Cancer Research Branch, Division of Cancer Biology, Research Institute, National Cancer Center, Goyang, Gyeonggi-do, Republic of Korea; University of Texas M. D. Anderson Cancer Center, UNITED STATES

## Abstract

Advances in oncology have enabled physicians to treat low-risk febrile neutropenia (FN) in outpatient settings. This study was aimed to explore the usefulness of the CISNE model and identify better triage in the emergency setting. This is a retrospective cohort study on 400 adult FN patients presenting to the Emergency Department of National Cancer Center, Korea from January 2010 to December 2016. All had been treated with cytotoxic chemotherapy for solid tumors in the previous 30 days. The primary outcome was the frequency of any serious complications during the duration of illness. Apparently stable patients numbered 299 (74.8%) of 400, and the remainder comprised clinically unstable patients. The stable patients fell into three cohorts according to the risk scores: CISNE I (low risk), 56 patients (18.7%); CISNE II (intermediate), 124 (41.5%) and CISNE III (high), 119 (39.8%). The primary outcome occurred in 10.7%, 19.4% and 33.6%, respectively, according to the cohort. Compared with the Multinational Association of Supportive Care in Cancer Risk Index Score (MASCC RIS), CISNE I stratum had significantly lower sensitivity (0.22 vs. 0.95 of MASCC low risk) but higher specificity (0.91 vs. 0.17) to predict zero occurrence of the primary outcome. The CISNE model was useful for identifying low-risk FN patients for outpatient treatment. The combination of the CISNE and MASCC RIS may help emergency physicians cope with FN more confidently.

## Introduction

### Background

As modern oncology progresses, it counts increasingly more on ambulatory care than on hospitalization. Most of the cytotoxic chemotherapy regimens are available at outpatient clinics as well, particularly for solid tumors. Febrile neutropenia (FN) remains one of the most common complications caused by therapy regardless of where it is provided. Additionally, FN related to bacteremia can bring on a high frequency of serious morbidity and mortality.[1] Since most cancer patients complaining of acute illnesses or breakthrough symptoms such as fever and pain visit the emergency department (ED) rather than outpatient clinics in Korea, emergency physicians are usually the first responders. An important issue in the optimal management strategy at the ED is where to continue the management chain. Several randomized controlled trials and meta-analysis have suggest that treatment with oral antibiotics in outpatient setting is as safe as inpatient treatment for a certain subset of patients at low risk.[2–7] Emergency physicians, therefore, need reliable triage tools to select the subset for outpatient treatment.

A risk stratification proposed by Multinational Association of Supportive Care in Cancer is considered a useful tool for the treatment of FN. The stratification, however, has some subjective elements such as the ‘burden of illness’, and it is devised to cover the entire spectrum of FN: fever during hospitalization as well as following intensive chemotherapy for hematologic malignancies. To predict low-risk FN, the MASCC RIS shows high sensitivity, but it has low specificity regarding solid tumors.[8–10]

The low specificity entails high false-positive rates to predict low-risk FN, leading ED physicians to take a risk of sending back some patients for outpatient treatment who should have been hospitalized. Consequently, the decision may jeopardize patient safety to an extent. Therefore, ED physicians may need a more reliable method that can identify low-risk FN.

### Objectives

This study was aimed to explore the usefulness of the CISNE model at the ED of a comprehensive cancer hospital in Korea. In addition, it compared the CISNE model with the MASCC RIS so that ED physicians can make an evidence-based decision regarding where to carry out treatment, outpatient vs. inpatient, and consequently maximize patient safety.

## Materials & methods

### Study design

**Fig 1:**
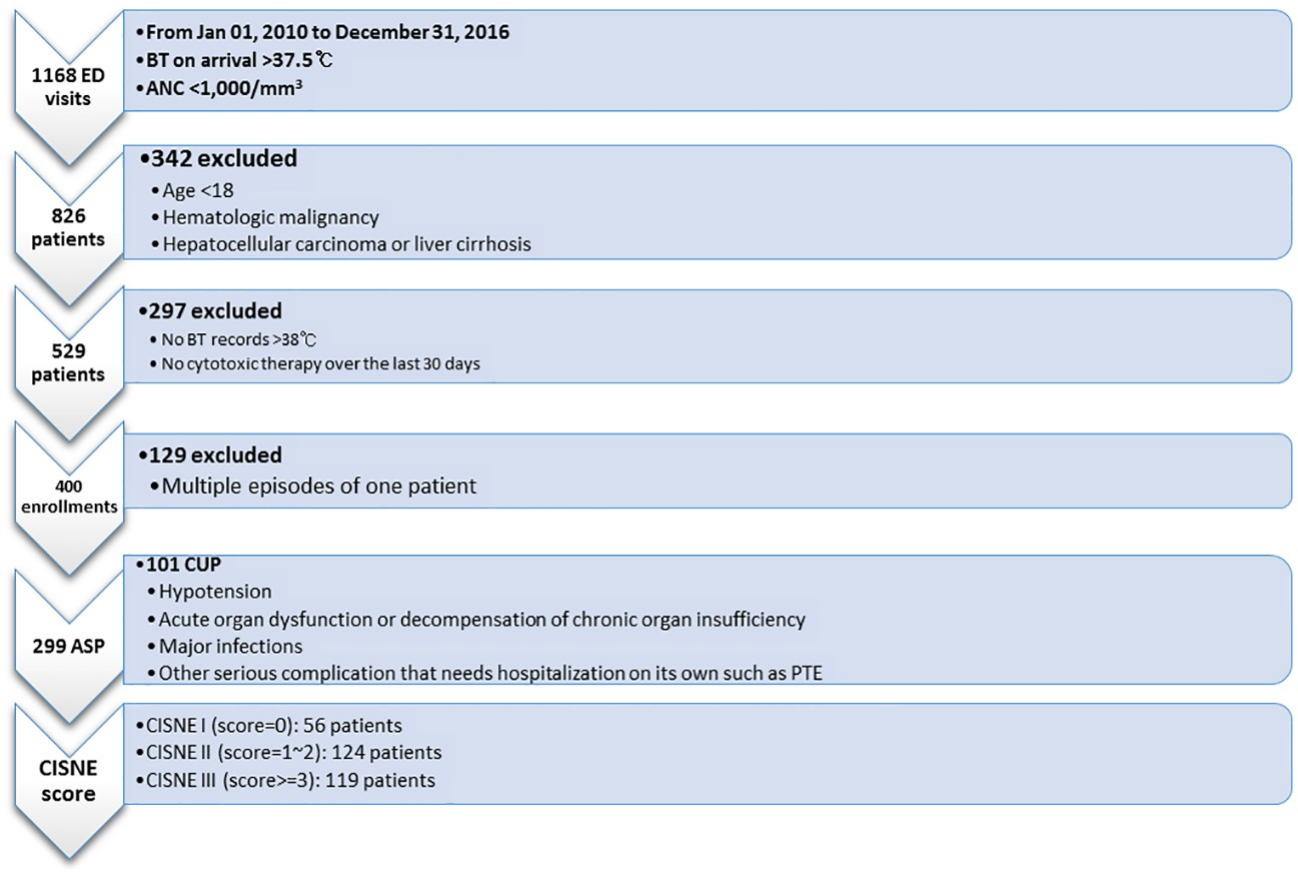
Study scheme. Two key conditions screened 1,168 patients who visited the emergency department from January 01, 2010 to December 31, 2016: a body temperature on arrival greater than 37.5°C and an absolute neutrophil count smaller than 1000/mm^3^. The study finally enrolled 400 patients for analysis after excluding those who met any of exclusion criteria as illustrated. BT, body temperature; ANC, absolute neutrophil count; PTE, pulmonary thromboembolism; ASP, apparently stable patients; CUP, clinically unstable patients.

The researchers screened FN patients who visited the ED of the National Cancer Center, Korea backward from December 31, 2016 to carry out a retrospective cohort study. Two key queries, equivalent to inclusion criteria, to search the institution’s database were as follows: 1) an absolute neutrophil count (ANC) less than 1,000/mm^3^ and 2) a body temperature recorded upon arriving at the ED greater than 37.5°C. Based on the results of previous studies, we planned to enroll approximately 450 FN episodes that would provide 360 Apparently Stable Patients (ASP).[10] A pilot investigation estimated the exclusion rate would be 50% if we applied the exclusion criteria as follows: 1) age younger than 18 years; 2) hematologic malignancy including lymphoma and multiple myeloma; 3) diagnosis of either hepatocellular carcinoma or liver cirrhosis; 4) no body temperature greater than 38°C, whether patient reported in 48 hours prior to his or her presentation to the ED or measured by healthcare professionals during the index visit; 5) no cytotoxic chemotherapeutics prescribed over the previous 30 days; and 6) the ANC level greater than 500/mm^3^ and not expected to further decrease below 500/mm^3^ over the next 48 hours. First, we aimed to extract consecutively at least 1,000 ED visits that fulfilled the inclusion criteria. The extraction, proceeding to the year of 2010, could retrieve 1,168 potentially eligible visits. The next step was to drop those that met any of the aforementioned exclusion criteria. Moreover, no other than the first episode was included if one patient visited the ED more than once for FN during the enrollment period. Therefore, the study intended to enroll adult FN patients presenting to the ED because of cytotoxic chemotherapy for solid tumors. Finally, we enlisted 400 cases and collected their data (Fig 1).

The institutional review board of the National Cancer Center, Korea approved this study (NCC2017-0239), which complied with the International Ethical Guidelines for Biomedical Research Involving Human Subjects, Good Clinical Practice Guidelines, the Declaration of Helsinki, and local rules and regulations. Additionally, the IRB waived the requirement for informed consent because of the study’s nature.

### Data collection

First, we classified the 400 patients into two groups: apparently stable patients (ASP) and clinically unstable patients (CUP).[10] ASP had neither acute organ dysfunction nor decompensation of chronic organ insufficiency, if any: heart, lung and kidney. Additionally, the group did not have hypotensive episodes during the index visit to ED, and any major infections were not suspected such as pneumonia, soft-tissue infection and biliary infection. Finally, ASP should not develop other complication(s) that would lead to hospitalization on its own—e.g., pulmonary thromboembolism, disseminated intravascular coagulopathy, acute arrhythmia, and major bleeding.

The study, reviewing electronic medical records, collected demographic, clinical, and laboratory data on ASP regardless of where the index episodes were treated: inpatient or outpatient. The duration of illness was defined as the days from the date of index visit to discharge in the case of hospitalization or, if the episode was managed at the outpatient clinics, to the last date of consultation sessions. Six variables were collected to yield CISNE scores: Eastern Cooperative Oncology Group performance status (2 points if ≥2), history of chronic obstructive pulmonary disease (1 point if present), history of chronic cardiovascular disease (1 point if present), oral mucositis (1 point if NCI grade ≥2), monocyte counts (1 point if <200/mm^3^) and serum glucose level to identify stress-induced hyperglycemia (2 points if present). Definitions of these variables were referenced in the FINITE study.[11] The sum brought 299 ASP to three cohorts: CISNE I or low risk if the sum was 0, CISNE II or intermediate risk if 1 or 2; CISNE III or high risk if ≥3. Clinical and laboratory findings relevant to the MASCC RIS were also reviewed, and the low-risk group was defined as 21 or greater. Any death as late as 7 days after the patients had left the hospital alive was also defined as mortality related to FN.

Any serious complications (ASC) were defined as positive if patients experienced one or more of the following: hypotension, acute heart dysfunction, acute kidney injury, acute respiratory failure, radiologic or endoscopic procedures to treat febrile illnesses such as percutaneous transhepatic biliary drainage, acute abdomen that needed urgent surgical or medical attention, major bleeding, disseminated intravascular coagulopathy, delirium or ICU care. If a patient experienced more than one of those, multiple counts were allowed.

### Outcome measures

The primary outcome was the frequency of ASC that occurred during the duration of illness. The secondary outcomes were 1) bacteremia 2) the composite of ASC or bacteremia and 3) frequency of individual serious complication.

### Statistical analysis

Categorical variables were summarized as frequencies and percentages, whereas medians and interquartile ranges (IQR) were calculated for continuous variables. To compare ASP vs. CUP as well as CISNE class I, II and III, we used chi-squared test for independence, Fisher’s exact test and the Cochrane-Armitage test for the trend, as appropriate, regarding categorical variables. T-tests or ANOVA was used for continuous variables if they showed normality. Otherwise, nonparametric methods (Mann-Whitney U test and Kruskal-Wallis test) were used. Receiver operating characteristic (ROC) analysis was performed to determine the MASCC and the CISNE model’s area under the curve (AUC) and compare with each other. Additionally, a logistic regression model was predefined to gauge the size of the effect with odds ratios to predict both serious complications (ASC) and the composite of ASC or bacteremia. Finally, we estimated the sensitivity and specificity of CISNE I and MASCC low risk with 95% confidence interval to predict zero occurrence of ASC. Across all analyses and tests, P-values less than 0.05 were considered as being statistically significant. Additionally, values less than 0.01 were represented as <0.01 for the sake of convenience. Statistical analysis for this paper was generated using the Real Statistics Resource Pack software [Release 4.3, copyright (2013–2015) Charles Zaiontz. www.real-statistics.com]. Additionally, MedCalc Statistical Software version 17.6 (MedCalc Software bvba, Ostend, Belgium; http://www.medcalc.org; 2017) was used to test the statistical significance of ROC analyses between independent variables and to perform Cochran-Armitage test for trend and logistic regression analyses.

## Results

### Apparently stable patients vs. clinically unstable patients

**Fig 2:**
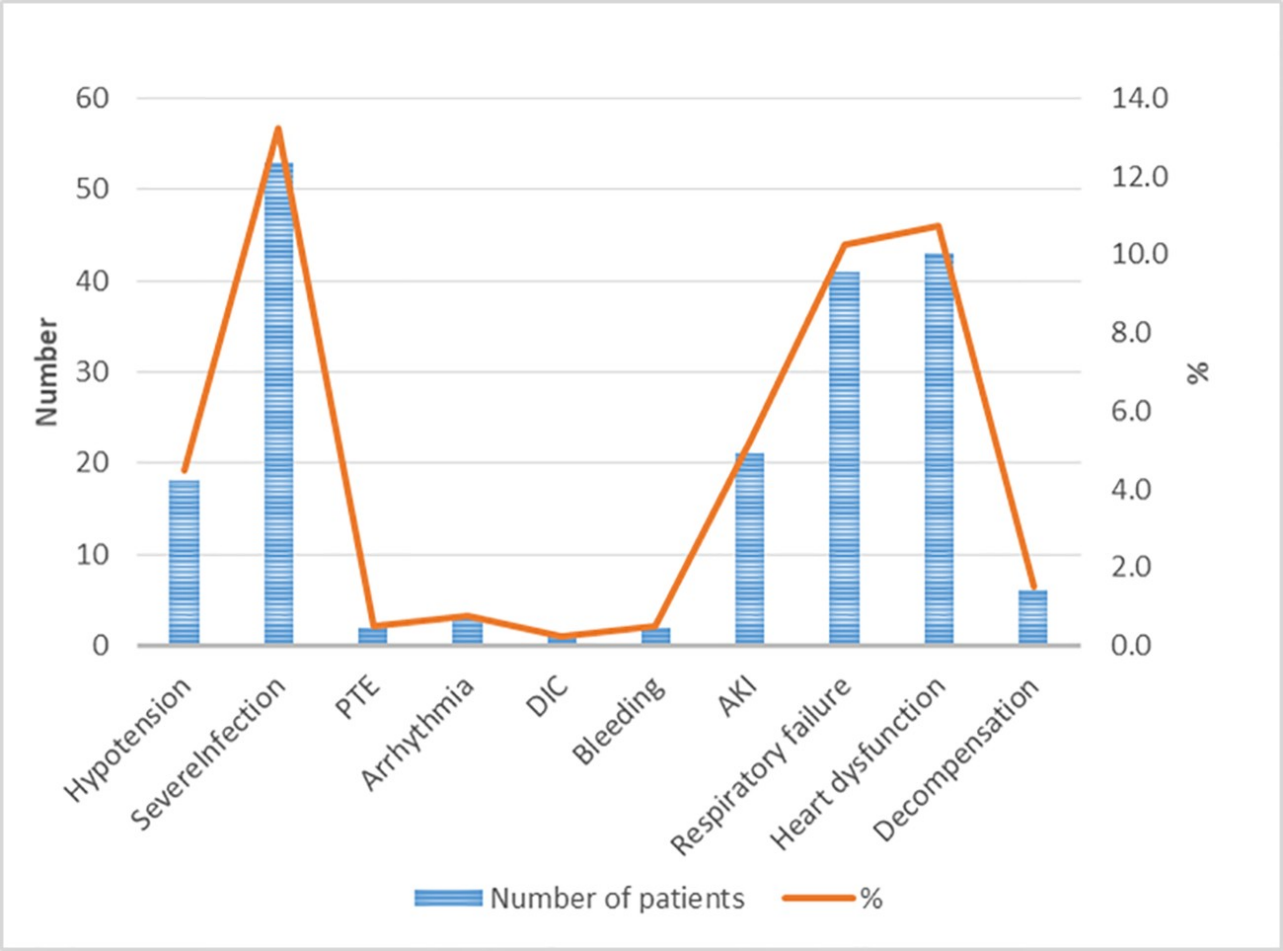
A chart showing the frequencies of acute complications that destabilized febrile neutropenia patients at the emergency department. % indicates the proportion of the entire patients (N = 400). “Decompensation” indicates decompensation of preexisting comorbidities. PTE, pulmonary thromboembolism; DIC, disseminated intravascular coagulopathy; AKI, acute kidney injury.

Table 1 describes the baseline characteristics of the entire study population (N = 400) while comparing ASP (N = 299) vs. CUP (N = 101). In line with its definition, CUP had more unfavorable factors than ASP. The former was more aged (64 vs. 52 years in ASP) and had more advanced cancer (stage IV cancer, 81.2 vs. 49.0%). Accordingly, the proportion of palliative chemotherapy was higher in CUP (74.3 vs. 32.4%). The median days elapsing from the latest chemotherapy to the index visit was 12 days (9–13, IQR) in ASP and 11 days (9–13, IQR) in CUP, and the difference was not statistically significant. Table 1 also demonstrates that the systolic blood pressures were significantly lower in CUP: 110 mmHg (100–126, IQR) vs. 120 mmHg (106.5–129, IQR) in ASP. At the ED, hypotension was recorded in 18 patients (4.5%), and serious infections that ED physicians diagnosed such as pneumonia brought 53 patients (13.3%) to CUP. The other events that automatically led patients to CUP were identified as follows: 43 patients with acute heart dysfunction (10.8%), 41 with acute respiratory failure (10.3%), 21 AKI (5.3%), and 6 patients with acute decompensation of preexisting chronic organ insufficiency. Fig 2 illustrates the distribution of those adverse events. Overall, 40 patients (10.0%) proved to have bacteremia (P-value <0.01). ED physicians decided outpatient treatment for 20 patients (5.0%), all of whom belonged to ASP. The median duration of illness was shorter in ASP, which was 4 days (3–7, IQR), than 10 days in CUP (6–17, IQR) and the difference was statistically significant.

**Table 1:** Baseline characteristics of the entire study population.

	Total (N = 400)	ASP (N = 299)	CUP (N = 101)	*P*
Median age, years (IQR)	56 (46–65)	52 (44–61)	64 (56–70)	<0.01
Female (%)	257 (64)	223 (74.6)	34 (33.7)	<0.01
Primary cancer				<0.01*
Breast (%)	148 (37)	134 (44.8)	14 (13.9)	
Gynecologic	58	50	8	
NSCLC	46	21	25	
SCLC	40	16	24	
Sarcoma	34	26	8	
Head & neck^a^	20	11	9	
Stomach	16	12	4	
Urologic	16	12	4	
Colorectal	9	7	2	
Pancreatobiliary	3	2	1	
Others^b^	9	7	2	
Stage ^c^ (%)				<0.01
I	42 (10.5)	41 (13.8)	1 (1.0)	
II	57 (14.3)	51 (17.1)	6 (5.9)	
III	72 (18.0)	60 (20.1)	12 (11.9)	
IV	228 (57.1)	146 (49.0)	82 (81.2)	
Treatment intent (%)				<0.01**
Palliative	172 (43)	97 (32.4)	75 (74.3)	
Adjuvant	152 (38.0)	138 (46.2)	14 (13.9)	
Neoadjuvant	53 (13.3)	49 (16.4)	4 (4.0)	
CCRT	23 (5.8)	15 (5.0)	8 (7.9)	
Median from chemotherapy to index visit to ED, days (IQR)	12 (9–13)	12 (9–13)	11 (9–13)	0.26
Median of systolic BP, mmHg (IQR)	118 (106.5–129)	120 (109.5–130)	110 (100–126)	<0.01
Hypotension ^d^ (%)	18 (4.5)	0	18 (17.8)	
Severe infection ^d^	53 (13.3)	0	53 (52.5)	
Acute organ failure ^d^(%)				
Heart	43 (10.8)	0	43 (42.6)	
Respiratory	41 (10.3)	0	41 (40.6)	
Renal	21 (5.3)	0	21 (20.8)	
Decompensation of chronic organ insufficiency ^d^ (%)	6 (1.5)	0	6 (5.9)	
Bacteremia (%)	40 (10.0)	17 (5.7)	23 (22.8)	<0.01
Discharge from ED (%)	20 (5.0)	20 (6.7)	0 (0)	
Median duration of illness, days (IQR)	5 (3–10)	4 (3–7)	10 (6–17)	<0.01
MASCC Low risk (%)	338 (84.5)	275 (92.0)	63 (62.4)	<0.01; *OR* = 6.9
Death (%)	21 (5.3)	0 (0)	21 (20.8)	

^a^ Including esophageal cancer

^b^ Including metastasis of unknown origin

^c^ One patient’s stage information is missing

^d^ All occurred in CUP as per definition

*Chi-square test for independence to compare breast *vs*. nonbreast cancer between the two groups

** Chi-square test for palliative *vs*. nonpalliative

NSCLC, nonsmall cell lung cancer; SCLC, small cell lung cancer; ED, emergency department; CCRT, concurrent chemoradiotherapy

In ASP, 275 patients (92.0%) belonged to the MASCC low-risk group (score sum ≥21), whereas 63 patients (62.4%) in CUP fell into the low-risk group; the difference was statistically significant (P-value <0.01). On the other hand, 21 patients (5.3%) died during the duration of illness, and all belonged to CUP.

### Clinical characteristics and outcomes according to CISNE triage

The study classified 299 ASP into three cohorts (CISNE I, II and III) based on their CISNE scores. Table 2 shows clinical and laboratory characteristics as well as major outcomes. The median ages were 49.5 years (42–57.3, IQR), 49.0 (4.0–58.0, IQR) and 58.0 (49.0–65.5, IQR) in CISNE I, II and III, respectively, and the difference was statistically significant. Diabetes mellitus was the most common comorbidity across the spectrum. Monocyte counts were significantly lower as the risk rose: 453/mm^3^ (337–653, IQR), 140 (50–312) and 40 (16–110) in CISNE I, II and III, respectively. By contrast, the severity of neutropenia, represented by the absolute neutrophil count, showed no statistically significant difference among the cohorts. The median serum glucose levels were 106 mg/dL (98–114, IQR), 116 (108–125) and 134 (125–154) in CISNE I, II and III, respectively, and there was statistical significance (P-value <0.01). The median of duration of illness was 3.5 days (2.0–5.0, IQR) in CISNE I, 4 days (3–6.3, IQR) in CISNE II and 5 days (4.0–9.0, IQR) in CISNE III; the differences were statistically significant (P-value <0.01). The frequency of hypotension, defined as a systolic blood pressure <90 mmHg, was not significantly different among the cohorts. Acute kidney injury (three cases) occurred exclusively in CISNE III, and the mean of the lowest estimated GFR was 95 ml/min (89.2–100.6; 95% CI), 93 (96.0–96.6; 95% CI) and 80 (76.4–84.2; 95% CI), respectively, which was statistically significant (P-value <0.01).

**Table 2:** Clinical characteristics and outcomes of 299 apparently stable patients according to CISNE triage: CISNE I (CISNE score = 0), II (score = 1 or 2), III (score ≥3). Statistical tests were not applied to some individual complications that occurred at very low frequency.

CISNE	I (N = 56, 18.7%)	II (N = 124, 41.5%)	III (N = 119, 39.8%)	*P*
Median age (IQR)	49.5 (42–57.3)	49 (40–58)	58 (49–65.5)	<0.01
Female (%)	51 (91.1)	96 (77.4)	76 (63.9)	<0.01
Comorbidities (%)	5 (8.9)	10 (8.1)	12 (10.1)	
COPD	0	0	0	
CCVD	0	1	2	
DM	5	9	10	
Median ANC (IQR)	95 (10–393)	24 (10–155)	20 (10–100)	0.16
Median of monocyte count	453 (337–635)	140 (50–312)	40 (16–110)	<0.01
Serum glucose, mg/dL (IQR)	106 (98–114)	116 (108–125)	134 (125–154)	<0.01
Median duration of illness, days (IQR)	3.5(2–5)	4 (3–6.3)	5 (4–9)	<0.01
Mean of lowest GFR, cc/min (95% C.I)	95 (89.2–100.6)	93 (89.0–96.6)	80 (76.4–84.2)	<0.01**
Any serious compli-cations (%)	6 (10.7)	24 (19.4)	40 (33.6)	<0.01*
Hypotension	6 (10.7)	15 (12.1)	21 (17.6)	0.16*
AKI	0	0	3 (2.5)	
AHD	0	1 (0.8)	4 (3.4)	
Arrhythmia	0	2 (1.6)	7 (5.9)	
ARF	0	2 (1.6)	7 (5.9)	
Acute abdomen	0	0	1 (0.8)	
Major bleeding	0	1 (0.8)	3 (2.5)	
DIC	0	0	1 (0.8)	
Delirium	0	7 (5.6)	9 (7.6)	
Invasive procedures	0	1 (0.8)	2 (1.7)	
Bacteremia (%)	2 (3.6)	6 (4.8)	9 (7.6)	0.25*
Composite of ASC or bacteremia (%)	7 (12.5)	29 (23.4)	43 (36.1)	<0.01*
MASCC score <21 (%)	3 (5.4)	5 (4.0)	16 (13.4)	0.02*

* Cochran-Armitage test for trend

** ANOVA

AKI, acute kidney injury; AHD, acute heart dysfunction; ARF, acute respiratory failure; ASC, any serious complications

Acute heart dysfunction and arrhythmia numbered 1 (0.8%) and 2 (3.4%) cases in CISNE II, respectively, and 4 (3.4%) and 7 (5.9%) cases in CISNE III, respectively. Acute respiratory failure also occurred in CISNE II (2 patients, 1.6%) and III (7, 5.9%). Seven (5.6%) patients in CISNE II and 9 (7.6%) in CISNE III experienced delirium. The remaining serious complications were relatively infrequent as described in Table 2.

The frequency of any serious complications, the primary outcome of this study, was 6 patients (10.7%), 24 patients (19.4%) and 40 patients (33.6%), in CISNE I, II and III, respectively, and the distribution was statistically significant (P-value <0.01). The composite outcome of any serious complications or bacteremia occurred in 7 patients (12.5%) of CISNE I, 29 (23.4%) of CISNE II and 43 (33.6%) of CISNE III. Additionally, there was a statistically significant trend among the three cohorts (P-value <0.01).

When applying the Multinational Association for Supportive Care in Cancer (MASCC) risk index to the study population, it was revealed that 3 patients (5.4%) of CISNE I, 5 (4.0%) of CISNE II and 16 (13.4%) of CISNE III belonged to the MASCC ‘High Risk’ group—that is, with an MASCC score <21 points.

### Comparison between the CISNE and MASCC models

**Fig 3:**
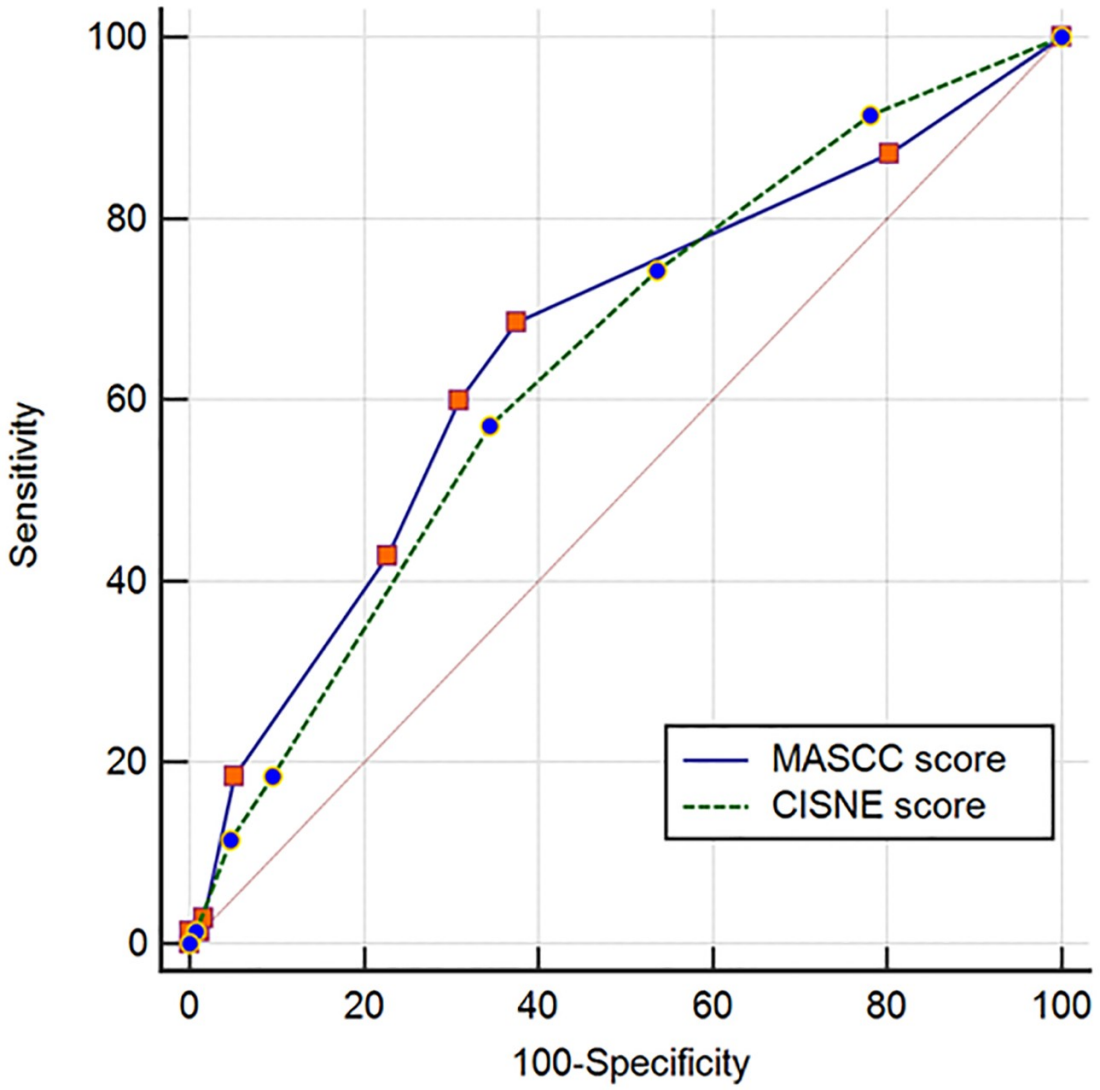
Receiver operating characteristic (ROC) analysis with respect to the MASCC score and CISNE score. Each variable’s AUC was 0.66 (95% CI; 0.60–0.71) and 0.64 (95% CI; 0.59–0.70), respectively. The difference between areas was 0.02 (95% CI;-0.08–0.11) and not statistically significant (P = 0.71).

We used ROC analysis and multiple logistic regression analysis to compare the two models. Fig 3 illustrates the two model’s ROC curves. With respect to the point estimate, the MASCC’s area under the curve was greater than CISNE: 0.66 vs. 0.64. However, the 95% confidence intervals were largely overlapping. Consequently, the difference between the two AUCs was 0.02 and not statistically significant (95% CI, -0.08~0.11; P-value = 0.71).

A multivariable logistic regression model was built using two predefined explanatory variables: the MASCC risk index and CISNE triage. Table 3 describes the results. Regarding any serious complications, the primary outcome, the MASCC category’s odds ratio, was 3.12 (95% CI, 1.30–7.49) and was statistically significant (P-value = 0.01). The CISNE triage’s odds ratio was 1.95 (95% CI, 1.29–2.94) and statistically significant (P-value <0.01). Regarding the composite outcome, both explanatory variables’ odds ratio decreased. The MASCC category’s odds ratio was 2.78 (95% CI, 1.17–6.62) and statistically significant (P-value = 0.02). The CISNE triage’s odds ratio was 1.74 (95% CI, 1.20–2.53) and statistically significant (P-value <0.01).

**Table 3:** Logistic regression analysis to predict any serious complications and composite of bacteremia or any serious complications.

	Any serious complications			The composite of any serious complications or bacteremia		
	OR (95% CI)	*P*-value	C-statistic	OR (95% CI)	*P*-value	C-statistic
MASCC	3.12 (1.30–7.49)	0.01	0.80	2.78 (1.17–6.62)	0.02	0.78
CISNE	1.95 (1.29–2.94)	<0.01		1.74 (1.20–2.53)	<0.01	

### Sensitivity and specificity to predict no serious outcome

We postulated that those who had no serious complications would be the subset at minimal risk even if ED physicians planned outpatient treatment. To identify which better fitted into the purpose, the study estimated and compared the sensitivity and specificity of CISNE I and the MASCC low-risk group. Table 4 shows the sensitivity and specificity of both tools. First, to predict no serious complications, CISNE I had low sensitivity (0.22, 95% CI; 0.17–0.28) but high specificity (0.91, 95% CI; 0.82–0.97). There was a stark contrast to the MASCC low-risk group: high sensitivity (0.95, 95% CI; 0.91–0.97) and low specificity (0.17, 95% CI; 0.09–0.28).

**Table 4:** Sensitivity and specificity of CISNE I and MASCC low risk in predicting no serious complications. The MASCC has high sensitivity but very low specificity. By contrast, the CISNE has high specificity but very low sensitivity.

	CISNE I	MASCC low risk
Sensitivity (95% CI)	0.22 (0.17–0.28)	0.95 (0.91–0.97)
Specificity (95% CI)	0.91 (0.82–0.97)	0.17 (0.09–0.28)

CISNE, Clinical Index of Stable Febrile Neutropenia; MASCC, Multinational Association of Supportive Care in Cancer

## Discussion

This study investigated 400 adult patients with FN presenting to the ED of a comprehensive tertiary cancer hospital while they underwent cytotoxic chemotherapy for various solid tumors. Approximately one-fourth of them, deemed to be CUP, had one or more reasons that demanded urgent medical stabilization on arrival (Table 1 and Fig 2). The remainder, by contrast, were “apparently stable” during the first few hours at ED. ASP, as expected, were less aged and had less advanced cancer in terms of TNM stage than CUP. Of interest, ASP were female-predominated largely because of the high proportion of breast cancer patients receiving adjuvant or neoadjuvant chemotherapy. ED physicians made a decision of outpatient treatment for 5% of patients, all of whom were ASP. On the other hand, the overall mortality was 5.3%, and all the fatal cases belonged to CUP, as well as those who needed ICU care for any reason. As such, these analyses hinted that the CUP criteria may well articulate physicians’ heuristics with simplicity. By contrast, MASCC low risk still accounted for 62.4% of CUP.

With respect to ASP, they were classified into three cohorts, namely, CISNE I (low risk), II (intermediate risk) and III (high risk). CISNE III patients were more aged and had a greater proportion of male gender. The differences in the absolute neutrophil count, systolic blood pressure and hypotension rates during illness were not statistically significant (the data sets are not presented). This finding contrasted with the other variables that showed statistically significant differences as the risk grew higher: monocyte count, serum glucose and estimated GFR.

The primary outcome, predefined as one or more complications, occurred in 10.7%, 19.4% and 33.6% in CISNE I, II and III, respectively, values that seemed to be higher than those in a recent study.[12] Notably, all six patients who experienced the primary outcome in CISNE I had nothing but hypotensive episodes, defined as any systolic blood pressure record below 90 mmHg. The episodes, nevertheless, did not cause symptoms and were treated at the general ward, with 300 to 500 mL of crystalloids rapidly infused as per the institution’s routine practice. No follow-up systolic blood pressure was lower than 90 mmHg following fluid infusion. Additionally, three of those events occurred in the night time, from 23:00 to 05:00. These observations brought us to a hypothesis that the three hypotensive events in CISNE I might be relevant to the diurnal variation of blood pressure that is physiologic.[13]

The distribution of bacteremia rates across the strata was not statistically significant in this study. The result is not in line with well-known evidence that bacteremia is a poor prognostic factor in FN.[14] The difference could be explained by two factors. First, our study’s sample size per stratum was too small to show a significant difference. Second, the study population was already deemed to be at low risk. We assume, therefore, that the impact of bacteremia might pale into insignificance as far as low-risk FN is concerned.

The key objective of this study is to determine how to identify patients that ED physicians may advise outpatient treatment rather than hospitalization at no expense to patient safety. In that regard, excellent predictors for discharge should have as low false-positive rates as possible, granting a concession to some “missed” low-risk patients who could have avoided unnecessary hospitalization. The statistics on Table 4 give us a hint that the two predictors, the MASCC RIS and CISNE, are perhaps a winning combination. The former has high sensitivity and low specificity; the latter is completely at odds. Moreover, a few studies demonstrated similar results: the complementary relationship between the MASCC RIS and CISNE.[12, 15, 16] The MASCC RIS seems to be suitable for ‘at a glance’ screening—that is, only a few elements ED physicians can quickly identify are sufficient for the triage. The physicians may hospitalize the patients at high risk whatever criteria they refer to, MASCC high risk or CUP, as proposed. Well-seasoned ED physicians are unlikely to feel a burden because unstable patients are usually obvious. Regarding “seemingly” stable patients, a final decision on hospitalization can be better guided by the CISNE than the MASCC RIS according to this study’s results.

This study has a few limitations. First, it was a retrospective study of a single institution’s experiences. Second, 95% of the study population was hospitalized, leading to a hypothesis that in-hospital supportive care, such as fluid therapy and clinical monitoring by healthcare professionals, would barely contribute to the outcomes of low-risk patients, compared with oral antibiotics. This study should be interpreted more conservatively if the hypothesis is to be denied.

## Conclusion

The CISNE model is useful for identifying low-risk FN patients with good clinical outcomes. Moreover, the combination of the CISNE and MASCC RIS models may be a good option to make a decision about FN patients at the ED. For example, ED practitioners can use the MASCC RIS to quickly screen out high-risk FN initially. Thereafter, to take a second look, they can calculate CISNE while planning outpatient treatment for low-risk FN.
